# Longer term impact of universal school-based mindfulness training compared with normal school provision on adolescent mental health and well-being, teacher mental health and school climate: results from the 2-year follow-up in the MYRIAD cluster randomised controlled trial

**DOI:** 10.1136/bmjment-2026-303016

**Published:** 2026-11-02

**Authors:** Benjamin Jones, Susan Ball, Louise Aukland, Ariane Beckley, Sarah-Jayne Blakemore, Sarah Byford, Tríona Casey, Catherine Crane, Tim Dalgleish, Nicola Dalrymple, Darren Dunning, Eleanor-Rose Farley, Tamsin Ford, Poushali Ganguli, Mark T Greenberg, Maria Kempnich, Liz Lord, Emma Medlicott, Jesus Montero-Marin, Elizabeth Nuthall, Lucy Palmer, Alice Phillips, Lucy Radley, Anam Raja, Laura Taylor, Alice Tickell, Obioha C Ukoumunne, Russell M Viner, Willem Kuyken, The MYRIAD Team, Saz Ahmed

**Affiliations:** 1 Department of Health and Community Sciences, Faculty of Health and Life Sciences, University of Exeter, National Institute for Health and Care Research Applied Research Collaboration South West, Exeter, UK; 2 Psychiatry Department, University of Oxford, Oxford, UK; 3 Experimental Psychology Department, University of Oxford, Oxford, UK; 4 Department of Psychology, University of Cambridge, Cambridge, UK; 5 UCL Institute of Cognitive Neuroscience, London, UK; 6 Health Service and Population Research Department, King’s College London, London, UK; 7 MRC Cognition and Brain Sciences Unit, Cambridge, UK; 8 Department of Health Sciences, University of York, York, UK; 9 Department of Psychiatry, University of Cambridge, Cambridge, UK; 10 Oxford Centre for Emerging Minds Research, University of Oxford, Oxford, UK; 11 Department of Human Development and Family Studies, Pennsylvania State University, Pennsylvania, Philadelphia, USA; 12 Teaching, Research and Innovation Unit, Parc Sanitari Sant Joan de Deu, Sant Boi de Llobregat, Spain; 13 Consortium for Biomedical Research in Epidemiology and Public Heatlh (CIBER Epidemiology and Public Health - CIBERESP), Madrid, Spain; 14 Offices of Clinical Research and the Provost, University of Texas Southwestern Medical Center, Dallas, Texas, USA; 15 Population, Policy and Practice Research Programme, UCL Great Ormond Street Institute of Child Health, London, UK

**Keywords:** Adolescent, Mental Health

## Abstract

**Background:**

The MY Resilience In ADolescence randomised controlled trial showed no superiority of universal school-based mindfulness training (SBMT) over social-emotional teaching as usual (TAU) regarding adolescents’ mental health and limited effects on teachers’ mental health and school climate at 1 year.

**Objective:**

Explore 2-year impact of SBMT versus TAU.

**Methods:**

85 UK schools (cohort 1: 975 pupils, 104 teachers; cohort 2: 7401 pupils, 575 teachers) randomised 1:1 to continue TAU (43 schools; 4232 pupils) or include SBMT (42 schools; 4144 pupils). The cohorts were analysed separately. Key pupil outcomes were risk for depression, social-emotional-behavioural functioning and well-being (Warwick-Edinburgh Mental Well-Being Scale (WEMWBS)). Teacher outcomes were burnout (Maslach Burnout Inventory-Educator Survey (MBI-ES)), self-efficacy, mindfulness, mindfulness in teaching (Mindfulness in Teaching Scale), stress (Perceived Stress Scale (PSS)), depression, anxiety and school climate.

Analyses used mixed-effects regression, allowing for correlation between observations from the same school and from the same class (pupil outcomes).

**Findings:**

SBMT was not superior to TAU in reducing pupil risk for depression (cohort 1: p=0.51; cohort 2: p=0.47) or social-emotional-behavioural functioning (cohort 1: p values ranged from 0.20 to 0.99; cohort 2: p values from 0.09 to 0.97). SBMT enhanced only cohort 2 pupils’ well-being: adjusted mean difference 1.0 (95% CI 0.2 to 1.8, p=0.02, WEMWBS).

SBMT did not impact cohort 1 teachers’ well-being (MBI-ES: exhaustion, p=0.19; depersonalisation, p=0.78; personal accomplishment, p=0.35), stress (PSS, p=0.68) or school climate (leadership and involvement, p=0.31; staff attitudes, p=0.09; respectful climate, p=0.87) but improved cohort 2 teachers’ well-being (personal accomplishment: −0.2, 95% CI −0.3 to −0.001, p=0.049), stress (−1.6, 95% CI −3.0 to −0.3, p=0.02) and school climate (leadership and involvement: 0.2, 95% CI 0.02 to 0.3, p=0.03; staff attitudes: 0.2, 95% CI 0.03 to 0.4, p=0.02; respectful climate: 0.2, 95% CI 0.1 to 0.4, p=0.001).

**Conclusions:**

Longer term outcomes broadly replicated 1-year findings, with little advantage of SBMT over TAU for pupils but some benefits for teacher-reported well-being and school climate persisting over time.

**Clinical implications:**

While social-emotional teaching in schools can be effective, with attention to wider context and individual differences, SBMT as delivered in this trial is not indicated for 11–14 year-olds. Small negative effects at 1 year washed out at year 2, and benefits for teachers largely persisted.

**Trial registration number:**

ISRCTN86619085.

**WHAT IS ALREADY KNOWN ON THIS TOPIC**

- Research suggests that school-based universal approaches to social-emotional learning offered to a whole population have the most potential to cost-effectively promote the mental health and well-being of young people.
- The MY Resilience In ADolescence cluster randomised controlled trial showed that universal school-based mindfulness training (SBMT) was not superior to social-emotional teaching as usual (TAU) in promoting adolescents’ mental health, but there were some positive effects of SBMT on teachers’ mental health and school climate at 1-year follow-up.

**WHAT THIS STUDY ADDS**

- This study explored the longer term impact of SBMT on pupils’ mental health and well-being, teachers’ mental health and school climate compared with TAU at 2-year follow-up.
- In the longer term, there is little advantage of SBMT over TAU for pupils, but the benefit of SBMT for teacher-reported well-being and school climate is maintained.

**HOW THIS STUDY MIGHT AFFECT RESEARCH, PRACTICE OR POLICY**

- Future research needs to evaluate potential indirect effects of teacher-reported improvements in well-being and school climate on pupils.
- School-based social-emotional teaching should focus on established mechanisms driving mental health at macro (eg, education policies), meso (eg, school climate), exo (eg, local context; relentless academic assessment that places pressure on schools, teachers and pupils) and micro (eg, peer relationships) levels, as well as codesigning interventions so as to maximise reach and engagement.

## Background

 There have been many calls to develop programmes for adolescents to reduce the risk of mental ill health and promote well-being.^1^ Because schools play a central role in the lives of the majority of children and families in the UK, they provide an opportune setting for promoting mental health and preventing mental health problems. Systematic reviews suggest that school-based universal approaches to social-emotional learning (SEL) offered to a whole population have the most potential to cost-effectively promote the mental health and well-being of young people.^2^ Mindfulness-based interventions (MBIs) are one such approach, aiming to teach foundational skills that can prevent depression and promote well-being. However, the evidence for universal MBIs in schools specifically is mixed, with typically small effects and substantial heterogeneity.^3^

Based on the premise that skills in attention and social-emotional-behavioural self-regulation underpin mental health and well-being across the full spectrum of well-being, we set up the MY Resilience In ADolescence (MYRIAD) programme of work (https://myriadproject.org/). This included a parallel-group, superiority cluster randomised controlled trial (RCT) examining the effectiveness of a school-based mindfulness training (SBMT) programme compared with social and emotional teaching as usual (TAU).^1^ 85 UK schools delivering TAU were recruited in two cohorts (cohort 1 in academic year 2016/2017; cohort 2 in 2017/2018). The cluster RCT showed no superiority of universal SBMT over TAU in promoting adolescents’ mental health,^4^ but some positive effects of SBMT on teachers’ mental health and school climate at 1-year follow-up.^5^ Subsequent secondary analyses showed that pupils reported very low engagement with home practice, and their views of SBMT were mixed.^6^ A recent scoping review, which included the MYRIAD trial, has raised the question of whether universal interventions might have adverse effects for some pupils.^7^ Two non-mutually exclusive mechanisms are offered. First, a universal, awareness-raising psychoeducation may prompt some higher risk pupils to attend more to distressing internal states without sufficient scaffolding to regulate them. However, the evidence for this specific mechanism in mindfulness/Cognitive Behavioural Therapy (CBT) interventions remains weak and is easily overgeneralised.^8^ Second, universal, one-size-fits-all delivery may be a poorer fit for pupils whose needs are qualitatively different and who may need more individualised or targeted support (consistent with Montero-Marin *et al*’s finding that pupils at higher risk of mental health problems showed lower responsiveness to and engagement with SBMT).^6^

Mirroring the broader literature, planned exploratory secondary analyses demonstrated positive effects on teachers’ well-being and school climate.^9 10^ The original primary endpoint specified in the trial protocol was the 2-year follow-up.^1^ The COVID-19 pandemic resulted in the change of primary endpoint from 2 years to 1 year, with 2 years retained as a key secondary endpoint.^2^ This was because all 1-year follow-up data were collected before the pandemic, but 2-year follow-up for participants in cohort 2 was delayed until approximately 30 months (ie, 6 months after the scheduled follow-up and still referred to as 2-year follow-ups). Despite this change, every effort was made to continue follow-up of pupils and teachers at 2 years.

### Objective

The study’s objective was to explore the longer term impact of SBMT on pupils’ mental health and well-being, teachers’ mental health and school climate, compared with TAU, through comparison of pupil- and teacher-level outcome measures at the 2-year follow-up.

## Methods

Reporting is in accordance with the Consolidated Standards of Reporting Trials (CONSORT) guidelines for cluster RCTs. Trial design and procedures are presented in full in the published protocol^1^ and update^2^ describing adaptations implemented due to the COVID-19 pandemic.

### Trial design, setting and participants

The MYRIAD trial was a parallel-group, superiority cluster RCT examining the effectiveness of an SBMT programme compared with TAU. The study assessed the effectiveness of SBMT on pupils and teachers. Key pupil outcomes were measures of risk for depression, social-emotional-behavioural functioning and well-being. Additional pupil and teacher outcomes measured mental health and school climate.

Because SBMT is a universal intervention delivered in schools, a cluster randomised design was appropriate, with randomisation at the school level. All mainstream secondary schools in the UK were eligible to take part, provided they had: (1) a substantive appointed head teacher; (2) not been judged inadequate in their most recent official inspection (eg, OFSTED); and (3) a strategy in place for delivery of adequate SEL curricula. Within schools, classes were subsampled, and all children in those classes were enrolled. Before randomisation, each school in the study nominated up to 15 staff members (teachers and others who were front-facing with the pupils; eg, support staff, reception staff and preferably on substantive contracts to minimise attrition) to participate. All nominated staff were eligible to complete personal mindfulness training, but only a maximum of five could be nominated by the school to progress to SBMT teacher training, and a smaller subset again was able to deliver the SBMT curriculum to their pupils (based on whether they could be scheduled to teach an eligible class), should their school be allocated to the SBMT arm.

Consent was sought from schools, before giving parents the opportunity to opt their children out of participation, and finally obtaining assent from the pupils themselves. Consent was also sought from participating teachers.

### Randomisation and allocation concealment

After baseline data collection, participating schools were randomised (1:1) to receive either SBMT or TAU by an independent researcher otherwise unconnected with the trial, using an allocation sequence generated by a similarly independent statistician. The randomisation balanced across the following prognostic factors: (1) school size (large (≥1000 pupils) vs small (<1000 pupils)); (2) school quality (‘outstanding/good’ vs ‘requires improvement’); and (3) deprivation level (below vs above the median percentage (29.4%) across UK schools of free school meal (FSM) eligibility, based on UK Department for Education data in 2017).^11^ For cohort 2 only, further balancing factors were introduced: school type (boys, girls, mixed) and region (England, Scotland, Wales, Northern Ireland). A constrained randomisation approach was implemented, where a sequence was selected ensuring a high degree of balance across the aforementioned factors.

Allocation concealment was ensured at the cluster level as schools were recruited before randomisation and allocated *en bloc* for each cohort, and at the individual participant level as pupils were recruited before schools were randomised. Due to the nature of the intervention, it was not possible to blind pupils or teachers to their school’s allocated group.

### Interventions

The SBMT programme and TAU are briefly outlined below and described in full in the protocol^1^ and earlier published work.^4^

The SBMT programme aims to teach mindfulness skills that support young people’s resilience using psychoeducation, class discussion and brief mindfulness practices. It is integrated into the school curriculum and taught by schoolteachers. SBMT involves 10 manualised, structured lessons (typically 30–50 min each), normally delivered over one school term, and home-based practices. The intention is to introduce young people to a range of skills (eg, attentional control, self-regulation of thoughts, feelings and behaviours) and encourage them to use these in their everyday lives. There are resources to support SBMT teachers (course booklets) and worksheets and online mindfulness practices for pupils.

The aim of the trial was to establish if SBMT adds value to current good SEL practice (TAU). SEL provision aims to prepare pupils with the knowledge, skills and attributes they need to manage their lives. It typically covers relationships, sex education and physical and mental health education. Relative to this broader SEL provision, SBMT teaches a narrower set of skills, focused specifically on mindfulness psychoeducation, guided in-class mindfulness practice and related discussion, rather than the wider range of topics (eg, broader self-care, relationships, explicit instruction in social problem solving) typically covered in SEL curricula. Schools randomised to SBMT could deliver it in addition to their existing SEL provision, partially in place of it, or instead of it; across participating classes, 52.4% used SBMT additively, 18.9% partially additively/substitutively and 23.1% substitutively for existing SEL teaching (delivery mode was not established for the remaining 5.6%). SEL provision was assessed by a researcher who was blind to the intervention allocation using a bespoke tool based in part on existing measures.

### Outcome measures

Teacher outcomes were collected pre-randomisation, after completion of personal mindfulness training and SBMT teacher training (preintervention), after delivery of SBMT to pupils (postintervention) and at 1 and 2 years after preintervention. Pupil outcomes were measured at the point of school consent (before randomisation), preintervention, postintervention and at 1 and 2 years after preintervention. When national lockdowns were introduced in the UK in March 2020, the 2-year follow-ups had been completed for pupils and teachers in cohort 1. However, due to lockdown restrictions, the 2-year follow-up in cohort 2 was delayed until approximately 30 months, although for ease of nomenclature it is still referred to as the 2-year follow-ups.

For pupils, the three key outcomes of interest at the 2-year follow-up were self-reported risk for depression using the Center for Epidemiologic Studies Depression Scale^12^; self-reported social-emotional-behavioural functioning using the Strengths and Difficulties Questionnaire (SDQ)^13^; and self-reported well-being using the Warwick-Edinburgh Mental Well-Being Scale (WEMWBS).^14^ Additional secondary pupil outcomes at 2 years include executive (dys)function using the Behavior Rating Inventory of Executive Function, Second Edition^15^; self-reported drug and alcohol use (measure designed for study); self-reported anxiety using the anxiety subscales from the Revised Child Anxiety and Depression Scale (RCADS)^16^; teacher-reported social-emotional-behavioural functioning using the teacher version of the SDQ; self-reported self-harm and suicidal ideation (measure designed for study); school climate using subscales from the School Climate and Connectedness Survey (SCCS)^17^; and self-reported mindfulness skills using the Child and Adolescent Mindfulness Measure.^18^

Teacher outcomes at the 2-year follow-up were burnout assessed using the Maslach Burnout Inventory-Educator Survey (MBI-ES),^19^ including exhaustion, depersonalisation and (lack of) personal accomplishment; self-efficacy using the Teachers’ Self-Efficacy Scale,^20^ including student engagement, instructional practice and classroom management; mindfulness using the Five Facet Mindfulness Questionnaire Short Form^21^; mindfulness in teaching using the Mindfulness in Teaching Scale,^22^ including interpersonal and intrapersonal mindfulness; stress using the Perceived Stress Scale (PSS)^23^; depression using the Patient Health Questionnaire 9^24^; anxiety using the Generalized Anxiety Disorder 7 assessment^25^; and school climate using the SCCS, including three scales deemed most relevant to SBMT: ‘School Leadership and Involvement’, ‘Staff Attitudes’ and ‘Respectful Climate’. Teacher engagement with the intervention was assessed according to whether they completed the mindfulness training and delivered SBMT sessions to pupils.

### Statistical analysis

Analyses followed a prespecified statistical analysis plan (SAP). The sample size calculation for the trial was based on the co-primary pupil outcomes at the 1-year follow-up and is reported in detail elsewhere.^2^ Consequently, no formal power calculations were undertaken for pupil or teacher outcomes at the 2-year follow-up.

As the timing of the 2-year follow-up differed between the two cohorts, as outlined above, all statistical analyses were undertaken separately for each cohort. Among 775 pupils in cohort 1 for whom data were available, the median (range) of timings of 2-year follow-ups was 25.2 months (22.1–27.6). For cohort 2, among 3042 pupils for whom data were available, the median (range) was 29.4 months (25.4–33.5).

Pupil, teacher and school characteristics were summarised using means and SDs for continuous variables and numbers and percentages for categorical variables. Missing outcome data were assumed to be missing at random and imputed using the multivariate linear mixed-effects model, with separate multiple imputation (MI) models constructed for each cohort. For pupil data, the cohort 1 MI model included outcomes at baseline (where available), preintervention and 2-year follow-up, as well as the stratification factors and gender dichotomised into ‘female’ versus ‘male and other’. The intention, as specified in the SAP, was to include outcome data at postintervention and 1-year follow-up, as well as utility scores (Child Health Utility 9D Index),^26^ but due to the small sample size in cohort 1, it was not possible to achieve model convergence. For cohort 2, outcome data at these time points were included in the MI model, in addition to utility scores; a second MI model for cohort 2, consistent with that used for cohort 1, was fitted for the purpose of a sensitivity analysis. For teachers, the MI models for each cohort included outcome data at all time points, trial arm status, the stratification factors, other characteristics prespecified for covariate adjustment and an indicator variable for completion of the full training/implementation route of SBMT. Sensitivity analyses using complete case data were also undertaken.

Continuous outcomes were compared between trial arms using mixed-effects linear regression and binary outcomes were analysed using marginal logistic regression models fitted with generalised estimating equations, with information sandwich (robust) estimates of the SE.^27^ These methods allow for the correlation between observations from the same school. The mixed-effects linear regression also allowed for an additional level of clustering at the class level (pupil outcomes only). The primary analysis of each outcome was adjusted for the stratification factors and the baseline value of the outcome, where available, in addition to gender (pupil outcomes only). Estimated effects and intracluster correlation coefficients were also obtained from unadjusted analyses.

Prespecified secondary analyses of teacher outcomes were undertaken using complier average causal effect (CACE) methods with instrumental variable regression to explore the causal effects of attendance at a minimum of four personal mindfulness training sessions, attendance at the 4-day training course and delivery of at least one mindfulness session to students. Teacher gender, years of teaching and preintervention stress measured using PSS were included in the first stage of the instrumental variable regression to predict ‘compliance’. The second stage included adjustment for the factors used to balance randomisation and the preintervention value of the outcome. Unadjusted mean differences, without adjustment at either stage, were also obtained.

All inferential results are presented with 95% CIs and p values. MI was undertaken using the *pan* package^28^ within R V.3.6.2, and analysis was undertaken using Stata V.17.0.

## Findings

Recruitment and follow-up are summarised in the flow diagrams in online supplemental figures 1–4, separately for pupils and teachers, and for each cohort. In total, 85 schools (13 cohort 1; 72 cohort 2) were recruited and randomised, comprising 8376 participating pupils (975 cohort 1; 7401 cohort 2) and 679 participating teachers (104 cohort 1; 575 cohort 2). 43 schools (7 cohort 1; 36 cohort 2) were allocated to SBMT, including 4232 pupils (551 cohort 1; 3681 cohort 2) and 362 teachers (58 cohort 1; 304 cohort 2). 42 schools (6 cohort 1; 36 cohort 2) comprising 4144 pupils (424 cohort 1; 3720 cohort 2) and 317 teachers (46 cohort 1; 271 cohort 2) were allocated to TAU. One school (TAU arm, cohort 1) withdrew soon after randomisation and before the period in which classes were selected for inclusion in the trial. This school was excluded from all summary statistics and analyses.

There was a markedly lower percentage follow-up in cohort 2 at the 2-year follow-up compared with cohort 1. In cohort 1, 81.3% (448/551) and 85.4% (362/424) of pupils who were allocated to the SBMT and TAU arm, respectively, were followed up at 2 years. In cohort 2, these follow-up percentages were 44.0% (1619/3681) and 39.9% (1484/3720). This difference can be attributed primarily to the COVID-19 pandemic, which changed and delayed data collection for participants in cohort 2. Nonetheless, pupil, teacher and school characteristics were similar across cohorts and allocated trial arms (table 1 and online supplemental table 1).

**Table 1:** Baseline characteristics of pupils in the two cohorts by trial arm status and overall

Pupil characteristics	Cohort 1			Cohort 2		
	SBMT	TAU	Total	SBMT	TAU	Total
	n=551	n=424	n=975	n=3681	n=3720	n=7401
Gender						
Female, n (%)	332 (60.4)	202 (47.8)	534 (54.9)	2018 (56.0)	1957 (53.8)	3975 (54.9)
Male, n (%)	204 (37.1)	211 (49.9)	415 (42.7)	1520 (42.1)	1612 (44.3)	3132 (43.2)
Other, n (%)	3 (0.6)	3 (0.7)	6 (0.6)	11 (0.3)	9 (0.3)	20 (0.3)
Prefer not to say, n (%)	11 (2.0)	7 (1.7)	18 (1.9)	58 (1.6)	62 (1.7)	120 (1.7)
Ethnicity—white, n (%)	460 (83.8)	337 (79.7)	797 (82.0)	2777 (77.2)	2628 (72.5)	5405 (74.9)
Age, mean (SD)	12.1 (0.6)	12.1 (0.6)	12.1 (0.6)	12.2 (0.6)	12.2 (0.6)	12.2 (0.6)
Year group						
Year 7, n (%)	308 (55.9)	271 (63.9)	579 (59.4)	1774 (48.2)	1871 (50.3)	3645 (49.3)
Year 8, n (%)	243 (44.1)	153 (36.1)	396 (40.6)	1828 (49.7)	1785 (48.0)	3613 (48.8)
Year 9, n (%)	–	–	–	79 (2.2)	64 (1.7)	143 (1.9)
Depression (CES-D), mean (SD)	13.8 (10.0)	13.4 (9.8)	13.6 (9.9)	13.5 (9.9)	13.3 (9.8)	13.4 (9.9)
Social-emotional-behavioural functioning (SDQ)						
Total difficulties—self-report, mean (SD)	11.9 (6.3)	11.0 (6.2)	11.5 (6.3)	11.8 (6.6)	11.8 (6.4)	11.8 (6.5)
Well-being (WEMWBS), mean (SD)	49.3 (10.4)	50.0 (9.4)	49.6 (10.0)	49.8 (9.6)	49.6 (9.8)	49.7 (9.7)

Cohort 1: sample size ranges from 549 to 551 pupils in the SBMT arm and 423 to 424 pupils in the TAU arm. In the SBMT arm, 550 pupils provided data on gender, SDQ and WEMWBS; 549 provided data on ethnicity. In the TAU arm, 423 pupils provided data on gender, ethnicity and SDQ. All 424 pupils in the TAU arm provided data on WEMWBS. Age, year group and CES-D data are available for all pupils.

Cohort 2: sample size ranges from 3596 to 3681 pupils in the SBMT arm and 3625 to 3720 pupils in the TAU arm. In the SBMT arm, 3607 pupils provided data on gender; 3596 provided data on ethnicity; and 3679 provided data on CES-D, 3621 on SDQ and 3664 on WEMWBS. In the TAU arm, 3640 pupils provided data on gender; 3625 provided data on ethnicity; and 3716 provided data on CES-D, 3658 on SDQ and 3695 on WEMWBS. Age and year group data are available for all pupils.

School year groups correspond across the home nations as follows: England—years 7, 8, 9 and 10; Northern Ireland—years 8, 9, 10 and 11.

CES-D, Center for Epidemiologic Studies Depression Scale; SBMT, school-based mindfulness training; SDQ, Strengths and Difficulties Questionnaire; TAU, teaching as usual; WEMWBS, Warwick-Edinburgh Mental Well-Being Scale.

### Pupils

Results from main (MI) and sensitivity (complete case) analyses comparing key (tables 2 and 3) and secondary (online supplemental tables 2 and 3) pupil outcomes between trial arms at 2 years are presented for cohort 1 and cohort 2. There was little evidence of a difference between trial arms for any of the three key pupil outcomes among cohort 1 pupils. For cohort 2 pupils, there was some evidence of a difference between trial arms in terms of well-being, with WEMWBS scores for pupils in the SBMT arm being, on average, higher than those in the TAU arm; estimated mean difference (SBMT−TAU) 1.0 (95% CI 0.2 to 1.8, p=0.02). There was little evidence of a difference between trial arms for the remaining two key pupil outcomes in cohort 2.

**Table 2:** Results from main analyses comparing key pupil outcomes between trial arms at 2 years: cohort 1

Outcome	SBMT Mean (SD)	TAU Mean (SD)	Unadjusted mean difference	Adjusted mean difference			ICC*
				Estimate	95% CI	P value	
Depression (CES-D)							
Multiple imputation†	n=444 18.2 (12.3)	n=362 16.6 (11.7)	1.4	0.7	−1.4 to 2.9	0.510	0.021
Complete case			1.3	0.8	−1.4 to 2.9	0.501	0.020
**Social-emotional-behavioural functioning (SDQ)—self-report**							
Total difficulties							
Multiple imputation†	n=444 13.6 (6.6)	n=362 12.9 (6.6)	0.6	0.2	−0.8 to 1.2	0.680	0.013
Complete case			0.6	0.3	−0.7 to 1.4	0.510	0.007
Emotional symptoms							
Multiple imputation†	n=444 4.3 (2.7)	n=362 3.9 (2.6)	0.3	0.2	−0.2 to 0.5	0.430	0.016
Complete case			0.3	0.2	−0.2 to 0.6	0.399	0.014
Conduct problems							
Multiple imputation†	n=444 2.3 (1.9)	n=362 2.3 (1.9)	0.02	0.003	−0.3 to 0.3	0.987	0.002
Complete case			0.03	0.1	−0.3 to 0.4	0.752	–
Hyperactivity/inattention							
Multiple imputation†	n=444 4.7 (2.6)	n=362 4.6 (2.6)	0.1	−0.1	−0.5 to 0.3	0.625	0.004
Complete case			0.1	−0.02	−0.4 to 0.4	0.914	0.000
Peer relationship problems							
Multiple imputation†	n=444 2.3 (1.9)	n=362 2.1 (1.7)	0.2	0.2	−0.1 to 0.5	0.202	0.017
Complete case			0.2	0.2	−0.03 to 0.5	0.083	0.009
Prosocial behaviour							
Multiple imputation†	n=444 7.4 (2.0)	n=362 7.3 (2.1)	0.1	−0.1	−0.4 to 0.2	0.653	0.003
Complete case			0.1	−0.1	−0.4 to 0.2	0.495	0.004
Well-being (WEMWBS)							
Multiple imputation†	n=448 47.1 (9.5)	n=361 47.4 (9.4)	−0.1	0.5	−1.2 to 2.2	0.560	0.023
Complete case			0.003	0.3	−1.4 to 2.0	0.733	0.020

n represents the number of students providing valid outcome data for each measurement.

* ICCs from unadjusted analyses.

† The multiple imputation model included only outcomes at baseline (where available), preintervention and at 2 years. Utility scores were excluded from the imputation model, and gender was dichotomised into ‘female’ versus ‘male & other’ for imputation and analysis.

CES-D, Center for Epidemiologic Studies Depression Scale; ICC, intracluster correlation coefficient; SBMT, school-based mindfulness training; SDQ, Strengths and Difficulties Questionnaire; TAU, teaching as usual; WEMWBS, Warwick-Edinburgh Mental Well-Being Scale.

**Table 3:** Results from main analyses comparing key pupil outcomes between trial arms at 2 years: cohort 2

Outcome	SBMT Mean (SD)	TAU Mean (SD)	Unadjusted mean difference	Adjusted mean difference			ICC*
				Estimate	95% CI	P value	
Depression (CES-D)							
Multiple imputation†	n=1603 20.4 (12.6)	n=1476 20.1 (12.3)	−0.1	−0.3	−1.3 to 0.6	0.465	0.023
Multiple imputation‡			−0.3	−0.5	−1.5 to 0.4	0.253	0.024
Complete case			0.2	−0.3	−1.5 to 0.9	0.629	0.037
**Social-emotional-behavioural functioning (SDQ)—self-report**							
Total difficulties							
Multiple imputation†	n=1583 14.5 (6.6)	n=1463 14.3 (6.6)	0.04	−0.03	−0.5 to 0.4	0.892	0.019
Multiple imputation‡			−0.1	−0.1	−0.6 to 0.4	0.733	0.021
Complete case			0.1	0.1	−0.6 to 0.7	0.864	0.028
Emotional symptoms							
Multiple imputation†	n=1583 4.7 (2.8)	n=1463 4.5 (2.7)	0.1	0.1	−0.1 to 0.3	0.474	0.024
Multiple imputation‡			0.1	0.1	−0.2 to 0.3	0.594	0.028
Complete case			0.2	0.1	−0.1 to 0.3	0.253	0.028
Conduct problems							
Multiple imputation†	n=1583 2.4 (1.9)	n=1463 2.3 (1.9)	0.03	0.003	−0.2 to 0.2	0.968	0.016
Multiple imputation‡			0.02	0.004	−0.2 to 0.2	0.970	0.023
Complete case			0.05	−0.01	−0.2 to 0.2	0.951	0.026
Hyperactivity/inattention							
Multiple imputation†	n=1583 5.0 (2.5)	n=1463 4.9 (2.5)	−0.003	0.02	−0.2 to 0.2	0.874	0.017
Multiple imputation‡			−0.02	0.005	−0.2 to 0.2	0.965	0.019
Complete case			0.02	0.1	−0.2 to 0.3	0.615	0.024
Peer relationship problems							
Multiple imputation†	n=1583 2.5 (1.8)	n=1463 2.6 (1.8)	−0.1	−0.1	−0.3 to 0.02	0.092	0.020
Multiple imputation‡			−0.2	−0.2	−0.3 to 0.02	0.073	0.022
Complete case			−0.1	−0.1	−0.3 to 0.1	0.202	0.019
Prosocial behaviour							
Multiple imputation†	n=1583 7.2 (2.1)	n=1463 7.2 (2.1)	0.1	0.01	−0.2 to 0.2	0.921	0.025
Multiple imputation‡			0.1	0.1	−0.1 to 0.3	0.506	0.024
Complete case			0.04	0.05	−0.1 to 0.2	0.645	0.019
Well-being (WEMWBS)							
Multiple imputation†	n=1615 45.0 (10.1)	n=1482 44.7 (9.6)	0.8	1.0	0.2 to 1.8	0.016	0.020
Multiple imputation‡			1.0	1.1	0.3 to 2.0	0.011	0.021
Complete case			0.5	0.8	−0.3 to 1.9	0.137	0.034

n represents the number of students providing valid outcome data for each measurement.

* ICCs from unadjusted analyses.

† The multiple imputation model included outcomes at baseline (where available), as well as all four post-baseline time points. Utility scores were included in the imputation model, and gender was maintained as three categories (‘female’, ‘male’ and ‘other’) for imputation and analysis.

‡ The multiple imputation model included only outcomes at baseline (where available), preintervention and at 2 years. Utility scores were excluded from the imputation model, and gender was dichotomised into ‘female’ versus ‘male & other’ for imputation and analysis.

CES-D, Center for Epidemiologic Studies Depression Scale; ICC, intracluster correlation coefficient; SBMT, school-based mindfulness training; SDQ, Strengths and Difficulties Questionnaire; TAU, teaching as usual; WEMWBS, Warwick-Edinburgh Mental Well-Being Scale.

Analyses of secondary pupil outcomes among cohort 1 showed evidence of a difference in only one outcome: the separation anxiety subscale of the RCADS (favouring TAU arm). SBMT pupils had, on average, scores that were higher than TAU pupils; estimated mean difference (SBMT−TAU) 0.8 (95% CI 0.03 to 1.6, p=0.04). There was little evidence of a difference between trial arms for any of the secondary outcomes among cohort 2 pupils. Results were similar under both MI models used for cohort 2 data for all outcomes.

Results remained broadly the same when analyses were repeated based on complete case data. For cohort 1, there were two exceptions for secondary outcomes: separation anxiety was no longer statistically significant—estimated mean difference (SBMT−TAU) 0.7 (95% CI −0.004 to 1.5, p=0.051); respectful climate showed a statistically significant difference between trial arms (favouring TAU arm)—estimated mean difference (SBMT−TAU) −0.2 (95% CI −0.3 to −0.04, p=0.007). For cohort 2, the effect of SBMT on WEMWBS was not statistically significant under the complete case analysis; estimated mean difference (SBMT−TAU) 0.8 (95% CI −0.3 to 1.9, p=0.14).

### Teachers

Results from main (MI), sensitivity (complete case) and additional (CACE) analyses of teacher outcomes at 2 years are presented in online supplemental tables 4 and 5 for cohort 1 and cohort 2, respectively. The main analyses showed little evidence of a difference between trial arms for any of the teacher outcomes for cohort 1. For cohort 2, there was evidence that SBMT teachers compared with TAU teachers had enhanced well-being according to the (lack of) personal accomplishment subscale of the MBI-ES (estimated mean difference −0.2, 95% CI −0.3 to −0.001, p=0.049); reduced stress as measured by the PSS (estimated mean difference −1.6, 95% CI −3.0 to −0.3, p=0.02); and reported improved school climate on all subscales of the SCCS (school leadership and involvement: estimated mean difference 0.2, 95% CI 0.02 to 0.3, p=0.03; staff attitudes: estimated mean difference 0.2, 95% CI 0.03 to 0.4, p=0.02; respectful climate: estimated mean difference 0.2, 95% CI 0.1 to 0.4, p=0.001). Results were broadly similar when analyses were repeated using complete case data. Exceptions for cohort 2 only were the estimated effect of SBMT on PSS, and on the school leadership and involvement and respectful climate subscales of the SCCS, all of which were no longer statistically significant under complete case analyses. Findings from CACE analyses of teacher outcomes were consistent with those from the main analyses for both cohorts.

## Discussion

### Summary and interpretation of findings

The current study broadly replicates the findings from our earlier study, which compared outcomes at the 1-year follow-up,^4^ and demonstrates that SBMT does not significantly differ from TAU in adolescent mental health outcomes over the longer term. Specifically, there were no substantial differences between the SBMT and TAU arms in terms of risk for depression, social-emotional-behavioural functioning or well-being in pupils at the 2-year follow-up. However, the small differences on a few secondary outcomes at 1-year follow-up showing some negative effects of SBMT compared with TAU^4^ had washed out.

These findings should be considered alongside the wider mixed literature on MBIs for youth mental health. Meta-analyses of universal school-based MBIs report small overall effect sizes for pupil depression and anxiety, particularly beyond 12 months, and a recent scoping review found that around 9% of school-based CBT and mindfulness trials report at least one negative outcome, most often concentrated on specific subgroups (eg, pupils with higher baseline mental health difficulties, male pupils, younger pupils and those eligible for FSM) rather than reflecting harm across the whole population.^7^ This pattern is broadly consistent with our own findings: no evidence of population-level harm from SBMT at 2 years alongside the possibility of small subgroup-specific effects. There is active debate about universal school mental health interventions. We would argue first that SEL programmes broadly are evidence based, especially with careful attention to implementation and fidelity. Second, population-level impacts of universal interventions can be disproportionate to their smaller per-pupil effect sizes, and current evidence of harm from SBMT, while important to take seriously, remains limited and should not be overgeneralised.^8^

For teachers and school climate, the results from the current study again broadly replicate those at 1-year follow-up.^5^ At the 2-year follow-up, SBMT was associated with slight improvements in teachers’ well-being and reductions in stress levels, as well as perceived improvements in school climate aspects such as school leadership, staff attitudes and respectful climate. Teachers’ well-being and school climate are important outcomes in their own right^7 9^; taken together, these findings suggest that SEL broadly and SBMT specifically might have important indirect effects on pupils, as well as longer term school (eg, culture) and teacher (eg, burnout, retention) outcomes.

Implementation and pupils’ subjective experience of SBMT are important context for these findings. In mixed-methods analyses of the MYRIAD trial, mean curriculum fidelity was 83.0% (SD 12.1), mean dose was 8.97 of 10 sessions (SD 2.1) and mean reach was 25.7% (SD 11.4) of the eligible year group school population; teachers’ baseline occupational stress moderated the effect of training intensity on implementation quality, such that more intensive training benefited implementation most among teachers who were already highly stressed.^6 9^ Between 70% and 90% of pupils reported practising mindfulness outside lessons ‘not at all’ to three times, and pupils’ responsiveness to SBMT was, on average, intermediate: around half rated the programme positively and half expressed reservations. Qualitative analyses of pupils’ open-ended responses identified both positive themes (eg, increased awareness of bodily sensations, improved ability to regulate emotions) and negative themes (eg, boredom, being prompted to notice difficult thoughts, feeling incompetent).^6^ Together, these findings suggest that implementation quality and pupils’ engagement with SBMT, rather than trial arm allocation alone, are likely to matter for whether pupils benefit; formal moderation analyses of pupil outcomes by school- or class-level implementation quality at 1- and 2-year follow-up were beyond the scope of the present study and are an important priority for future analysis of the MYRIAD dataset.

### Strengths and limitations

The key strength of this study is that it adds further findings from the MYRIAD cluster RCT, which is the largest evaluation of the effectiveness of the SBMT programme in early adolescence on mental health and well-being, by evaluating outcomes over the longer term. The MYRIAD study was originally designed with the 2-year follow-up as the primary endpoint. Due to the COVID-19 pandemic, the primary endpoint was changed to the 1-year follow-up, with the 2-year follow-up retained as a key secondary endpoint. Despite the disruption caused by COVID-19, this 2-year follow-up was completed (although delayed in cohort 2) and has provided a valuable opportunity to evaluate the longer term impact of SBMT compared with TAU on pupils, teachers and school climate. There was good retention of pupils at follow-up in cohort 1 (83%). On the other hand, retention in cohort 2, whose follow-up was impacted by the COVID-19 pandemic, was lower, with 42% of randomised pupils followed up at 2 years. Teacher retention was 52% in cohort 1 and 49% in cohort 2. Further study limitations include the following: the use of adolescent self-reported secondary outcomes—however, this was mitigated by including teacher-reported versions of several outcomes (eg, social-emotional-behavioural functioning); by necessity, pupils and teachers were not blind to treatment allocation; and finally, the study was powered based on planned single analyses of the two cohorts combined, rather than separate analyses by cohort. The separate analyses, deemed most appropriate due to the delayed follow-ups for cohort 2, have resulted in smaller than anticipated sample sizes and consequent reductions in precision.

### Clinical implications

Systematic reviews and meta-analyses suggest that universal social-emotional teaching can improve pupils’ mental health outcomes, especially with careful attention to implementation.^29^

However, both this and our earlier study suggest that universal SBMT, delivered as we have done, is not indicated for young adolescents, although no adverse effects persisted at the 2-year follow-up. In our review and exploratory analyses trying to make sense of the findings, we have argued that going forward, school-based social-emotional teaching should focus on established mechanisms driving mental health at macro (eg, education policies), meso (eg, school climate), exo (eg, local context; relentless academic assessment that places pressure on schools, teachers and pupils) and micro (eg, peer relationships) levels, as well as codesigning interventions so as to maximise reach and engagement.^6 30^

The potential benefit of SBMT for teachers’ mental health and school climate at 2 years seen in the current study, which could indirectly benefit pupils, means that future research should evaluate these potential indirect effects.

Schools and policy-makers considering SBMT should weigh this balance of evidence, attend to implementation quality and consider an approach best supported by evidence of pupil, teacher and school climate benefits, such as well-implemented SEL provision.

## Supplementary material

10.1136/bmjment-2026-303016 online supplemental file 1

## Data Availability

Data are available upon reasonable request.
