# Supplementary material for: Longer term impact of universal school-based mindfulness training compared with normal school provision on adolescent mental health and well-being, teacher mental health and school climate: results from the 2-year follow-up in the MYRIAD cluster randomised controlled trial
Source: BMJ Ment Health. 2026 Nov 2;29(1):e303016. doi: 10.1136/bmjment-2026-303016 (PMC13735371; doi:10.1136/bmjment-2026-303016)
Supplement: Supplementary file 1 [file bmjment-29-1-s001.docx]

**Supplementary material**

**Supplementary Figure S1:** Pupil flow (Cohort 1 - all data collected prior to COVID-19)

Pre-intervention: Autumn term 2017

Post-intervention: Summer Term 2018

one-year follow-up: Autumn Term 2018

**Intervention delivered between January and July**

2nd academic year (2017/18)

3^rd^ academic year (2018/19)

4^th^ academic year (2019/20)

two-year follow-up: Autumn Term 2019

**COVID-19 Outbreak**

**Schools consented to participate**

K = 13 schools

N = 975 pupils

Mean (SD) 81 (13.8) pupils per school*

**Allocated to SBMT**

K = 7 schools

N = 551 pupils

Mean (SD) 79 (15.7) pupils per school

**Allocated to TAU**

K = 6 schools

N = 424 pupils

Mean (SD) 85 (11.5) pupils per school**

K = 7 schools

N = 523 pupils

Mean (SD) 75 (15.5) pupils per school

Baseline: Autumn term 2016

K = 5 schools

N = 400 pupils

Mean (SD) 80 (10.1) pupils per school

K = 7 schools

N = 474 pupils

Mean (SD) 68 (19.3) pupils per school

K = 7 schools

N = 477 pupils

Mean (SD) 68 (17.2) pupils per school

K = 5 schools

N = 395 pupils

Mean (SD) 79 (13.5) pupils per school

K = 5 schools

N = 387 pupils

Mean (SD) 77 (13.4) pupils per school

**Missing**

schools K = 0

pupils N = 28

**Missing**

schools K = 1

pupils N = 24

**Missing**

schools K = 0

pupils N = 77

**Missing**

schools K = 1

pupils N = 37

**Missing**

schools K = 0

pupils N = 74

**Missing**

schools K = 1

pupils N = 29

1st academic year (2016/17)

K = 7 schools

N = 448 pupils

Mean (SD) 64 (15.3) pupils per school

K = 5 schools

N = 362 pupils

Mean (SD) 72 (15.4) pupils per school

**Missing**

schools K = 0

pupils N = 103

**Missing**

schools K = 1

pupils N = 62

Note: ‘Missing’ is the number of schools (K) / students (N) that did not provide data on any of the three primary outcomes at the subsequent timepoint. Students could be temporarily lost to follow-up if not in school for a given timepoint. For the TAU school that dropped out soon after randomisation, no pupils were included in the trial and so no data were provided*.*

*Summary statistics based on K = 12 schools, given one school dropped out soon after randomisation and before recruiting and pupils.

**Summary statistics based on K = 5 schools, given one school dropped out soon after randomisation and before recruiting and pupils.

SD: standard deviation. SBMT: school-based mindfulness training. TAU: teaching as usual.

**Supplementary Figure S2:** Pupil flow (Cohort 2 – one-year follow-up data collected prior to COVID-19, two-year follow-up data collected after COVID-19)

Pre-intervention: Autumn term 2018

Post-intervention: Summer Term 2019

one-year follow-up: Autumn Term 2019

**Intervention delivered between January and July**

2nd academic year (2018/19)

3^rd^ academic year (2019/20)

4^th^ academic year (2020/21)

two-year follow-up: Summer Term 2021

**COVID-19 Outbreak**

**Schools consented to participate**

K = 72 schools

N = 7401 pupils

Mean (SD) 103 (21.8) pupils per school

**Allocated to SBMT**

K = 36 schools

N = 3681 pupils

Mean (SD) 102 (28.0) pupils per school

**Allocated to TAU**

K = 36 schools

N = 3720 pupils

Mean (SD) 103 (13.5) pupils per school

K = 36 schools

N = 3554 pupils

Mean (SD) 99 (27.2) pupils per school

Baseline: Autumn term 2017

K = 36 schools

N = 3591 pupils

Mean (SD) 100 (13.9) pupils per school

K = 36 schools

N = 3305 pupils

Mean (SD) 92 (25.9) pupils per school

K = 36 schools

N = 3201 pupils

Mean (SD) 89 (24.3) pupils per school

K = 36 schools

N = 3403 pupils

Mean (SD) 95 (17.3) pupils per school

K = 36 schools

N = 3185 pupils

Mean (SD) 88 (15.9) pupils per school

**Missing**

schools K = 0

pupils N = 127

**Missing**

schools K = 0

pupils N = 129

**Missing**

schools K = 0

pupils N = 376

**Missing**

schools K = 0

pupils N = 535

**Missing**

schools K = 0

pupils N = 480

**Missing**

schools K = 0

pupils N = 317

1st academic year (2017/18)

K = 29 schools

N = 1619 pupils

Mean (SD) 56 (26.3) pupils per school

K = 25 schools

N = 1484 pupils

Mean (SD) 59 (27.8) pupils per school

**Missing**

schools K = 7

pupils N = 2062

**Missing**

schools K = 12

pupils N = 2236

Note: ‘Missing’ is the number of schools (K) / students (N) that did not provide data on any of the three primary outcomes at the subsequent timepoint. Students could be temporarily lost to follow-up if not in school for a given timepoint.

SD: standard deviation. SBMT: school-based mindfulness training. TAU: teaching as usual.

**Supplementary Figure S3:** Teacher flow (Cohort 1 - all data collected prior to COVID-19)

Pre-intervention: Autumn term 2017

Post-intervention: Summer Term 2018

one-year follow-up: Autumn Term 2018

**Intervention delivered between January and July**

2nd academic year (2017/18)

3^rd^ academic year (2018/19)

4^th^ academic year (2019/20)

two-year follow-up: Autumn Term 2019

**COVID-19 Outbreak**

**Schools consented to participate**

K = 13 schools

N = 104 teachers

**Allocated to SBMT**

K = 7 schools

N = 58 teachers

Mean (SD) teachers per school

8.3 (2.2)

**Allocated to TAU**

K= 6 schools

N = 46 teachers

Mean (SD) teachers per school

7.7 (1.6)

K = 7 schools

N = 40 teachers

Mean (SD) teachers per school 8.3 (2.2)

Baseline: Autumn term 2016

K = 5 schools

N = 30 teachers

Mean (SD) teachers per school 7.8 (1.8)

K = 7 schools

N = 31 teachers

Mean (SD) teachers per school 8.3 (2.2)

K= 7 schools

N = 27 teachers

Mean (SD) teachers per school 8.3 (2.2)

K = 5 schools

N = 24 teachers

Mean (SD) teachers per school 7.8 (1.8)

K= 5 schools

N = 20 teachers

Mean (SD) teachers per school 7.8 (1.8)

**Missing**

schools K = 0

teachers N = 18

**Missing**

schools K = 1

teachers N = 16

**Missing**

schools K = 0

teachers N = 27

**Missing**

schools K = 1

teachers N = 26

**Missing**

schools K = 0

teachers N = 31

**Missing**

schools K = 1

teachers N = 22

1st academic year (2016/17)

K = 7 schools

N = 31 teachers

Mean (SD) teachers per school 8.3 (2.2)

K = 5 schools

N = 23 teachers

Mean (SD) teachers per school 7.8 (1.8)

**Missing**

schools K = 0

teachers N = 27

**Missing**

schools K = 1

teachers N = 23

Note: ‘Missing’ is the number of schools (K) / teachers (N) that did not provide data on any outcomes at the subsequent timepoint. Teachers could be temporarily lost to follow-up if not in school for a given timepoint.

SD: standard deviation. SBMT: school-based mindfulness training. TAU: teaching as usual.

**Supplementary Figure S4:** Teacher flow (Cohort 2 – one-year follow-up data collected prior to COVID-19, two-year follow-up data collected after COVID-19)

Pre-intervention: Autumn term 2018

Post-intervention: Summer Term 2019

one-year follow-up: Autumn Term 2019

**Intervention delivered between January and July**

2nd academic year (2018/19)

3^rd^ academic year (2019/20)

4^th^ academic year (2020/21)

two-year follow-up: Summer Term 2021

**COVID-19 Outbreak**

**Schools consented to participate**

K = 72 schools

N = 575 teachers

**Allocated to SBMT**

K= 36 schools

N = 304 teachers

Mean (SD) teachers per school 8.4 (2.2)

**Allocated to TAU**

K = 36 schools

N = 271 teachers

Mean (SD) teachers per school 7.5 (2.1)

K = 36 schools

N = 229 teachers

Mean (SD) teachers per school 8.4 (2.2)

Baseline: Autumn term 2017

K = 36 schools

N = 192 teachers

Mean (SD) teachers per school 7.5 (2.1)

K = 35 schools

N = 178 teachers

Mean (SD) teachers per school 8.5 (2.1)

K = 36 schools

N = 187 teachers

Mean (SD) teachers per school 8.4 (2.2)

Mean (SD) teachers per school 8.5 (2.1)

K= 36 schools

N = 161 teachers

Mean (SD) teachers per school 7.5 (2.1)

K = 34 schools

N = 152 teachers

Mean (SD) teachers per school 7.7 (2.1)

**Missing**

schools K = 0

teachers N = 75

**Missing**

schools K = 0

teachers N = 79

**Missing**

schools K = 1

teachers N = 126

**Missing**

schools K = 2

teachers N = 119

**Missing**

schools K = 0

teachers N = 117

**Missing**

schools K = 0

teachers N = 110

1st academic year (2017/18)

K = 36 schools

N = 155 teachers

Mean (SD) teachers per school 8.4 (2.2)

K = 33 schools

N = 125 teachers

Mean (SD) teachers per school 7.7 (2.1)

**Missing**

schools K = 0

teachers N = 149

**Missing**

schools K = 3

teachers N = 146

Note: ‘Missing’ is the number of schools (K) / teachers (N) that did not provide data on any outcomes at the subsequent timepoint. Teachers could be temporarily lost to follow-up if not in school for a given timepoint.

SD: standard deviation. SBMT: school-based mindfulness training. TAU: teaching as usual.

**Supplementary Table S1:** Baseline characteristics of schools and teachers in the two cohorts by trial arm status and overall

|  |  | **Cohort 1** | | | | |  | **Cohort 2** | | | | |
| --- | --- | --- | --- | --- | --- | --- | --- | --- | --- | --- | --- | --- |
|  |  | **SBMT** |  | **TAU** |  | **Total** |  | **SBMT** |  | **TAU** |  | **Total** |
| **School characteristics** |  | **K = 7** |  | **K = 6** |  | **K = 13** |  | **K = 36** |  | **K = 36** |  | **K = 72** |
| Region |  |  |  |  |  |  |  |  |  |  |  |  |
| England, n (%) |  | 7 (100) |  | 6 (100) |  | 13 (100) |  | 31 (86) |  | 31 (86) |  | 62 (86) |
| Scotland, n (%) |  | - |  | - |  | - |  | 1 (3) |  | 2 (6) |  | 3 (4) |
| Wales, n (%) |  | - |  | - |  | - |  | 2 (6) |  | 1 (3) |  | 3 (4) |
| Northern Ireland, n (%) |  | - |  | - |  | - |  | 2 (6) |  | 2 (6) |  | 4 (6) |
| School size – at least 1000 pupils, n (%) |  | 3 (43) |  | 3 (50) |  | 6 (46) |  | 17 (47) |  | 20 (56) |  | 37 (51) |
| Type of school |  |  |  |  |  |  |  |  |  |  |  |  |
| Mixed, n (%) |  | 6 (86) |  | 6 (100) |  | 12 (92) |  | 30 (83) |  | 32 (89) |  | 62 (86) |
| Girls, n (%) |  | 1 (14) |  | 0 (0) |  | 1 (8) |  | 6 (17) |  | 4 (11) |  | 10 (14) |
| School quality rating |  |  |  |  |  |  |  |  |  |  |  |  |
| Requires improvement, n (%) |  | 1 (14) |  | 1 (17) |  | 2 (15) |  | 5 (14) |  | 4 (11) |  | 9 (13) |
| Does not require improvement, n (%) |  | 6 (86) |  | 5 (83) |  | 11 (85) |  | 31 (86) |  | 32 (89) |  | 63 (88) |
| Deprivation |  |  |  |  |  |  |  |  |  |  |  |  |
| Above median (29.4%) for free school meals, n (%) |  | 1 (14) |  | 1 (17) |  | 2 (15) |  | 14 (39) |  | 14 (39) |  | 28 (39) |
| Below median (29.4%) for free school meals, n (%) |  | 6 (86) |  | 5 (83) |  | 11 (85) |  | 22 (61) |  | 22 (61) |  | 44 (61) |
|  |  |  |  |  |  |  |  |  |  |  |  |  |
| **Teacher characteristics** |  | **N = 58** |  | **N = 46** |  | **N = 104** |  | **N = 304** |  | **N = 271** |  | **N = 575** |
| Age, mean (SD) |  | 38.7 (8.7) |  | 37.9 (8.9) |  | 38.3 (8.8) |  | 40.5 (8.9) |  | 39.4 (9.3) |  | 40.0 (9.1) |
| Gender |  |  |  |  |  |  |  |  |  |  |  |  |
| Female, n (%) |  | 43 (74.1) |  | 34 (73.9) |  | 77 (74.0) |  | 240 (79.2) |  | 189 (69.7) |  | 429 (74.7) |
| Male, n (%) |  | 15 (25.9) |  | 12 (26.1) |  | 27 (26.0) |  | 62 (20.5) |  | 81 (29.9) |  | 143 (24.9) |
| Other, n (%) |  | - |  | - |  | - |  | 1 (0.3) |  | 1 (0.4) |  | 2 (0.4) |
| Ethnicity |  |  |  |  |  |  |  |  |  |  |  |  |
| Arab/Arab British, n (%) |  | - |  | - |  | - |  | 0 (0.0) |  | 2 (0.7) |  | 2 (0.4) |
| Asian/British Asian, n (%) |  | 1 (1.7) |  | 2 (4.4) |  | 3 (2.9) |  | 6 (2.0) |  | 11 (4.1) |  | 17 (3.0) |
| Black/African/Caribbean, n (%) |  | 1 (1.7) |  | 0 (0.0) |  | 1 (1.0) |  | 1 (0.3) |  | 3 (1.1) |  | 4 (0.7) |
| Mixed/Multiple Ethnic Group, n (%) |  | 2 (3.5) |  | 1 (2.2) |  | 3 (2.9) |  | 7 (2.3) |  | 3 (1.1) |  | 10 (1.7) |
| White, n (%) |  | 54 (93.1) |  | 43 (93.5) |  | 97 (93.3) |  | 287 (94.7) |  | 249 (91.9) |  | 536 (93.4) |
| Other Ethnic Group, n (%) |  | - |  | - |  | - |  | 2 (0.7) |  | 3 (1.1) |  | 5 (0.9) |
| Qualified teacher status, n (%) |  | 55 (94.8) |  | 45 (97.8) |  | 100 (96.2) |  | 298 (98.4) |  | 264 (97.4) |  | 562 (97.9) |
| Years teaching experience, mean (SD) |  | 10.8 (7.9) |  | 11.8 (8.3) |  | 11.3 (8.1) |  | 13.3 (8.6) |  | 12.3 (8.7) |  | 12.8 (8.6) |
| Experience of mindfulness |  |  |  |  |  |  |  |  |  |  |  |  |
| Currently practicing mindfulness, n (%) |  | 3 (5.2) |  | 2 (4.4) |  | 5 (4.8) |  | 36 (11.9) |  | 19 (7.0) |  | 55 (9.6) |
| Some understanding of mindfulness, n (%) |  | 44 (75.9) |  | 38 (82.6) |  | 82 (78.9) |  | 247 (81.5) |  | 230 (84.9) |  | 477 (83.1) |
| Not aware of mindfulness, n (%) |  | 11 (19.0) |  | 6 (13.0) |  | 17 (16.4) |  | 20 (6.6) |  | 22 (8.1) |  | 42 (7.3) |
| Maslach Burnout Inventory (MBI Educators Survey) |  |  |  |  |  |  |  |  |  |  |  |  |
| Emotional exhaustion, mean (SD) |  | 2.3 (1.2) |  | 2.4 (1.1) |  | 2.4 (1.2) |  | 2.4 (1.2) |  | 2.3 (1.2) |  | 2.4 (1.2) |
| Depersonalisation, mean (SD) |  | 0.7 (0.7) |  | 1.0 (1.1) |  | 0.9 (0.9) |  | 0.7 (0.7) |  | 0.7 (0.8) |  | 0.7 (0.8) |
| Personal accomplishment, mean (SD) |  | 1.0 (0.6) |  | 1.2 (0.8) |  | 1.1 (0.7) |  | 1.0 (0.7) |  | 1.0 (0.7) |  | 1.0 (0.7) |
| Teachers' Sense of Efficacy Scale (TSES) |  |  |  |  |  |  |  |  |  |  |  |  |
| Student engagement, mean (SD) |  | 6.9 (1.0) |  | 6.6 (1.1) |  | 6.7 (1.1) |  | 6.9 (1.1) |  | 6.8 (1.0) |  | 6.9 (1.1) |
| Instructional practice, mean (SD) |  | 7.3 (1.0) |  | 7.3 (0.9) |  | 7.3 (1.0) |  | 7.3 (0.9) |  | 7.4 (1.0) |  | 7.4 (1.0) |
| Classroom management, mean (SD) |  | 7.5 (1.0) |  | 7.5 (1.0) |  | 7.5 (1.0) |  | 7.6 (0.9) |  | 7.5 (1.0) |  | 7.6 (1.0) |
| Five Facet Mindfulness Questionnaire (FFMQ-SF), mean (SD) |  | 83.7 (12.8) |  | 81.2 (12.4) |  | 82.6 (12.7) |  | 83.6 (12.6) |  | 85.3 (13.4) |  | 84.4 (13.0) |
| Mindfulness in Teaching Scale (MTS) |  |  |  |  |  |  |  |  |  |  |  |  |
| Interpersonal, mean (SD) |  | 20.2 (2.4) |  | 20.8 (2.3) |  | 20.5 (2.4) |  | 20.7 (2.5) |  | 20.6 (2.6) |  | 20.6 (2.6) |
| Intrapersonal, mean (SD) |  | 33.0 (5.7) |  | 32.4 (5.4) |  | 32.8 (5.6) |  | 33.5 (5.1) |  | 34.2 (5.6) |  | 33.8 (5.3) |
| Perceived Stress Scale (PSS), mean (SD) |  | 16.0 (7.2) |  | 16.9 (7.4) |  | 16.4 (7.3) |  | 16.5 (6.9) |  | 16.4 (7.1) |  | 16.4 (7.0) |
| Patient Health Questionnaire (PHQ-9), mean (SD) |  | 5.0 (4.3) |  | 5.8 (5.2) |  | 5.3 (4.7) |  | 5.2 (4.3) |  | 5.1 (4.5) |  | 5.2 (4.4) |
| Generalized Anxiety Disorder - 7 (GAD-7), mean (SD) |  | 4.2 (4.1) |  | 5.0 (5.2) |  | 4.5 (4.6) |  | 4.3 (4.0) |  | 4.6 (4.4) |  | 4.5 (4.2) |
| School Climate and Connectedness Survey (SCCS) |  |  |  |  |  |  |  |  |  |  |  |  |
| School leadership and involvement, mean (SD) |  | 3.9 (0.5) |  | 3.9 (0.8) |  | 3.9 (0.7) |  | 3.9 (0.7) |  | 3.9 (0.6) |  | 3.9 (0.6) |
| Staff attitudes, mean (SD) |  | 4.2 (0.5) |  | 4.2 (0.6) |  | 4.2 (0.5) |  | 4.1 (0.6) |  | 4.2 (0.6) |  | 4.1 (0.6) |
| Respectful climate, mean (SD) |  | 3.8 (0.5) |  | 3.8 (0.6) |  | 3.8 (0.5) |  | 3.8 (0.6) |  | 3.8 (0.6) |  | 3.8 (0.6) |

*Cohort 1*: Data on provision of PSHE are provided for all 7 schools in the SBMT arm and 5 schools in the TAU arm. All schools in both arms provided data on the remaining characteristics.

Sample size ranges from 57 to 58 teachers in the SBMT arm and is 46 teachers in the TAU arm. In the SBMT arm, 57 teachers provided data on SCCS school leadership and involvement and staff attitudes subscales. All teachers in the SBMT arm provided data on the remaining characteristics. All teachers in the TAU arm provided data on all characteristics.

*Cohort 2:* All schools in both arms provided data on school characteristics. Sample size ranges from 302 to 304 teachers in the SBMT arm and from 270 to 271 teachers in the TAU arm. In the SBMT arm, 302 teachers provided data on MTS subscales, 303 teachers provided data on age, gender, ethnicity, qualified teacher status, years teaching experience, experience of mindfulness, PSS, PHQ-9, GAD-7, FFMQ-SF and TSES subscales. Data on SCCS and MBI subscales were provided by all teachers in the SBMT arm. In the TAU arm, 270 teachers provided data on age, MBI and MTS subscales, PSS, PHQ-9, GAD-7, FFMQ-SF and TSES subscales. Data on gender, ethnicity, qualified teacher status, years teaching experience, experience of mindfulness and SCCS subscales were provided by all teachers in the TAU arm.

SBMT: school-based mindfulness training. TAU: teaching as usual. PSHE: Personal, Social, Health and Economic Education. SD: standard deviation

**Supplementary Table S2:** Results from main analyses comparing secondary pupil outcomes between trial arms at two-years: Cohort 1

| **Outcome** | **SBMT** | **TAU** | **Unadjusted** | **Adjusted mean difference /**  **odds ratio^a^** | | | **ICC^b^** |
| --- | --- | --- | --- | --- | --- | --- | --- |
|  | **mean (SD) / n (%)** | **mean (SD) / n (%)** | **mean difference / odds ratio^a^** | **estimate** | **95% CI** | **p-value** |  |
| Executive processing (BRIEF2) – self-report |  |  |  |  |  |  |  |
| Multiple Imputation^1^ | N = 387  85.7 (22.8) | N = 349  83.8 (22.4) | 2.0 | 1.0 | -3.4 to 5.5 | 0.647 | 0.008 |
| Complete Case |  |  | 1.7 | 1.3 | -3.1 to 5.6 | 0.563 | 0.000 |
| Drug use – self-report |  |  |  |  |  |  |  |
| Multiple Imputation^1^ | 94/407 (23.1%) | 67/350 (19.1%) | 1.3 | 1.3 | 0.9 to 1.8 | 0.197 | 0.002 |
| Complete Case |  |  | 1.3 | 1.2 | 0.9 to 1.5 | 0.248 | -0.003 |
| Alcohol use – self-report |  |  |  |  |  |  |  |
| Multiple Imputation^1^ | 266/415 (64.1%) | 197/356 (55.3%) | 1.4 | 1.4 | 0.9 to 2.1 | 0.136 | 0.025 |
| Complete Case |  |  | 1.4 | 1.4 | 0.9 to 2.0 | 0.098 | 0.024 |
| **Anxiety (RCADS) – self-report** |  |  |  |  |  |  |  |
| Total score |  |  |  |  |  |  |  |
| Multiple Imputation^1^ | N = 422  32.3 (23.0) | N = 359  28.3 (20.6) | 4.2 | 3.4 | -1.2 to 7.9 | 0.143 | 0.010 |
| Complete Case |  |  | 3.8 | 2.8 | -1.1 to 6.8 | 0.162 | 0.001 |
| Social phobia |  |  |  |  |  |  |  |
| Multiple Imputation^1^ | N = 422  11.4 (6.7) | N = 359  10.6 (6.7) | 0.8 | 0.5 | -0.7 to 1.7 | 0.388 | 0.002 |
| Complete Case |  |  | 0.8 | 0.4 | -0.8 to 1.5 | 0.501 | 0.000 |
| Panic disorder |  |  |  |  |  |  |  |
| Multiple Imputation^1^ | N = 422  7.1 (6.7) | N = 359  5.9 (6.3) | 1.3 | 1.0 | -0.1 to 2.2 | 0.081 | 0.005 |
| Complete Case |  |  | 1.2 | 0.8 | -0.3 to 2.0 | 0.143 | 0.000 |
| Separation anxiety |  |  |  |  |  |  |  |
| Multiple Imputation^1^ | N = 422  3.5 (3.9) | N = 359  2.6 (2.9) | 0.9 | 0.8 | 0.03 to 1.6 | 0.042 | 0.016 |
| Complete Case |  |  | 0.8 | 0.7 | -0.004 to 1.5 | 0.051 | 0.008 |
| Generalised anxiety |  |  |  |  |  |  |  |
| Multiple Imputation^1^ | N = 422  6.2 (4.4) | N = 359  5.5 (4.2) | 0.6 | 0.6 | -0.4 to 1.5 | 0.246 | 0.014 |
| Complete Case |  |  | 0.6 | 0.5 | -0.3 to 1.4 | 0.231 | 0.006 |
| Obsessive-compulsive |  |  |  |  |  |  |  |
| Multiple Imputation^1^ | N = 423  4.2 (4.1) | N = 359  3.7 (3.6) | 0.5 | 0.5 | -0.4 to 1.4 | 0.282 | 0.014 |
| Complete Case |  |  | 0.4 | 0.4 | -0.4 to 1.2 | 0.327 | 0.005 |
| Self-harm – self-report |  |  |  |  |  |  |  |
| Multiple Imputation^1^ | 67/406 (16.5%) | 43/319 (13.5%) | 1.2 | 1.2 | 0.7 to 2.1 | 0.494 | 0.010 |
| Complete Case |  |  | 1.3 | 1.2 | 0.7 to 2.1 | 0.614 | 0.003 |
| Suicide ideation – self-report |  |  |  |  |  |  |  |
| Multiple Imputation^1^ | 104/399 (26.1%) | 78/314 (24.8%) | 1.0 | 1.0 | 0.7 to 1.6 | 0.960 | 0.022 |
| Complete Case |  |  | 1.0 | 1.0 | 0.6 to 1.6 | 0.950 | 0.018 |
| Mindfulness skills (CAMM) – self-report |  |  |  |  |  |  |  |
| Multiple Imputation^1^ | N = 436  25.0 (8.7) | N = 359  26.1 (9.2) | -1.2 | -0.8 | -2.5 to 0.9 | 0.343 | 0.007 |
| Complete Case |  |  | -0.9 | -0.6 | -2.5 to 1.2 | 0.517 | 0.010 |
| **School ecology/climate (SCCS**) |  |  |  |  |  |  |  |
| School Leadership and Student Involvement |  |  |  |  |  |  |  |
| Multiple Imputation^1^ | N = 430  3.0 (0.9) | N = 359  3.1 (0.8) | -0.2 | -0.2 | -0.5 to 0.1 | 0.240 | 0.053 |
| Complete Case |  |  | -0.2 | -0.2 | -0.5 to 0.1 | 0.266 | 0.053 |
| Respectful Climate |  |  |  |  |  |  |  |
| Multiple Imputation^1^ | N = 430  3.1 (0.8) | N = 358  3.3 (0.8) | -0.2 | -0.1 | -0.3 to 0.004 | 0.056 | 0.005 |
| Complete Case |  |  | -0.2 | -0.2 | -0.3 to -0.04 | 0.007 | 0.000 |
| Peer Climate |  |  |  |  |  |  |  |
| Multiple Imputation^1^ | N = 429  2.9 (0.7) | N = 359  3.0 (0.8) | -0.1 | -0.1 | -0.4 to 0.1 | 0.236 | 0.042 |
| Complete Case |  |  | -0.1 | -0.1 | -0.4 to 0.1 | 0.203 | 0.045 |
| Caring Adults |  |  |  |  |  |  |  |
| Multiple Imputation^1^ | N = 429  3.2 (0.9) | N = 359  3.2 (0.9) | -0.04 | -0.1 | -0.2 to 0.1 | 0.535 | 0.004 |
| Complete Case |  |  | -0.03 | -0.1 | -0.2 to 0.1 | 0.593 | 0.004 |

^1^ The multiple imputation model included only outcomes at baseline (where available), pre-intervention and at two-years. Utility scores were excluded from the imputation model, and gender was dichotomized into “female” versus “male & other” for imputation and analysis.

^a^ Odds ratios are presented for the outcomes: drug use (any drug use versus no drug use), alcohol use (any alcohol use versus no alcohol use), self harm (“yes” & ”prefer not to say” versus “no”) and suicide ideation (“yes” & “prefer not to say” versus “no”).

^b^ ICCs from unadjusted analyses.

Note: The Ns presented in the table represent the number of students providing valid outcome data for each measurement.

SBMT: school-based mindfulness training. TAU: teaching as usual. ICC: intra-cluster correlation coefficient. SD: standard deviation. CI: confidence interval. BRIEF2: Behaviour Rating Inventory of Executive Function, Second edition. RCADS: Revised Child Anxiety and Depression Scale. CAMM: Child-and Adolescent Mindfulness Measure. SCCS: School Climate and Connectedness survey.

**Supplementary table S3:** Results from main analyses comparing secondary pupil outcomes between trial arms at two-years: Cohort 2

| **Outcome** | **SBMT** | **TAU** | **Unadjusted** | **Adjusted mean difference /**  **odds ratio^a^** | | | **ICC^b^** |
| --- | --- | --- | --- | --- | --- | --- | --- |
|  | **mean (SD) / n (%)** | **mean (SD) / n (%)** | **mean difference / odds ratio^a^** | **estimate** | **95% CI** | **p-value** |  |
| Executive processing (BRIEF2) – self-report |  |  |  |  |  |  |  |
| Multiple Imputation^1^ | N = 1354  88.2 (23.4) | N = 1362  87.5 (22.6) | 0.05 | -0.3 | -2.1 to 1.5 | 0.766 | 0.013 |
| Multiple Imputation^2^ |  |  | -0.4 | -0.6 | -2.4 to 1.2 | 0.503 | 0.016 |
| Complete Case |  |  | 0.6 | 0.2 | -2.0 to 2.5 | 0.845 | 0.019 |
| Drug use – self-report |  |  |  |  |  |  |  |
| Multiple Imputation^1^ | 226/1411 (16.0%) | 250/1347 (18.6%) | 0.9 | 0.9 | 0.6 to 1.2 | 0.377 | 0.033 |
| Multiple Imputation^2^ |  |  | 0.9 | 0.9 | 0.6 to 1.2 | 0.443 | 0.040 |
| Complete Case |  |  | 0.8 | 0.8 | 0.6 to 1.1 | 0.108 | 0.020 |
| Alcohol use – self-report |  |  |  |  |  |  |  |
| Multiple Imputation^1^ | 658/1425 (46.2%) | 658/1351 (48.7%) | 0.9 | 0.9 | 0.7 to 1.2 | 0.409 | 0.078 |
| Multiple Imputation^2^ |  |  | 0.9 | 0.9 | 0.7 to 1.2 | 0.475 | 0.081 |
| Complete Case |  |  | 0.9 | 0.9 | 0.7 to 1.3 | 0.740 | 0.088 |
| **Anxiety (RCADS) – self-report** |  |  |  |  |  |  |  |
| Total score |  |  |  |  |  |  |  |
| Multiple Imputation^1^ | N = 1421  36.0 (22.6) | N = 1406  34.2 (22.0) | 0.7 | 0.4 | -1.2 to 2.1 | 0.623 | 0.026 |
| Multiple Imputation^2^ |  |  | 0.7 | 0.4 | -1.4 to 2.3 | 0.647 | 0.027 |
| Complete Case |  |  | 2.1 | 1.5 | -0.5 to 3.5 | 0.144 | 0.035 |
| Social phobia |  |  |  |  |  |  |  |
| Multiple Imputation^1^ | N = 1426  12.6 (7.3) | N = 1406  12.3 (7.0) | -0.1 | -0.2 | -0.7 to 0.4 | 0.522 | 0.042 |
| Multiple Imputation^2^ |  |  | -0.1 | -0.2 | -0.8 to 0.5 | 0.628 | 0.047 |
| Complete Case |  |  | 0.4 | 0.3 | -0.5 to 1.1 | 0.418 | 0.062 |
| Panic disorder |  |  |  |  |  |  |  |
| Multiple Imputation^1^ | N = 1421  7.9 (6.6) | N = 1406  7.3 (6.4) | 0.4 | 0.3 | -0.2 to 0.8 | 0.231 | 0.022 |
| Multiple Imputation^2^ |  |  | 0.4 | 0.3 | -0.2 to 0.9 | 0.243 | 0.023 |
| Complete Case |  |  | 0.7 | 0.5 | -0.1 to 1.1 | 0.095 | 0.028 |
| Separation anxiety |  |  |  |  |  |  |  |
| Multiple Imputation^1^ | N = 1428  3.9 (3.7) | N = 1406  3.6 (3.6) | 0.3 | 0.2 | -0.1 to 0.5 | 0.176 | 0.015 |
| Multiple Imputation^2^ |  |  | 0.2 | 0.2 | -0.1 to 0.5 | 0.281 | 0.017 |
| Complete Case |  |  | 0.3 | 0.2 | -0.1 to 0.6 | 0.159 | 0.019 |
| Generalized anxiety |  |  |  |  |  |  |  |
| Multiple Imputation^1^ | N = 1428  6.8 (4.5) | N = 1406  6.5 (4.4) | 0.04 | -0.01 | -0.4 to 0.4 | 0.967 | 0.025 |
| Multiple Imputation^2^ |  |  | 0.05 | 0.002 | -0.4 to 0.4 | 0.991 | 0.027 |
| Complete Case |  |  | 0.4 | 0.3 | -0.1 to 0.6 | 0.162 | 0.025 |
| Obsessive-compulsive |  |  |  |  |  |  |  |
| Multiple Imputation^1^ | N = 1428  4.7 (4.1) | N = 1406  4.5 (3.9) | 0.1 | 0.05 | -0.3 to 0.4 | 0.779 | 0.011 |
| Multiple Imputation^2^ |  |  | 0.1 | 0.1 | -0.3 to 0.4 | 0.713 | 0.011 |
| Complete Case |  |  | 0.3 | 0.2 | -0.1 to 0.6 | 0.231 | 0.009 |
| Self-harm – self-report |  |  |  |  |  |  |  |
| Multiple Imputation^1^ | 228/1463 (15.6%) | 185/1312 (14.1%) | 1.0 | 1.0 | 0.8 to 1.4 | 0.856 | 0.020 |
| Multiple Imputation^2^ |  |  | 1.0 | 1.0 | 0.7 to 1.3 | 0.936 | 0.026 |
| Complete Case |  |  | 1.1 | 1.1 | 0.8 to 1.3 | 0.664 | 0.010 |
| Suicide ideation – self-report |  |  |  |  |  |  |  |
| Multiple Imputation^1^ | 449/1408 (31.9%) | 391/1272 (30.7%) | 1.0 | 1.0 | 0.8 to 1.3 | 0.979 | 0.029 |
| Multiple Imputation^2^ |  |  | 1.0 | 1.0 | 0.8 to 1.3 | 0.928 | 0.032 |
| Complete Case |  |  | 1.1 | 1.0 | 0.8 to 1.3 | 0.944 | 0.034 |
| Mindfulness skills (CAMM) – self-report |  |  |  |  |  |  |  |
| Multiple Imputation^1^ | N = 1516  23.7 (9.5) | N = 1433  24.4 (9.2) | -0.4 | -0.3 | -1.1 to 0.5 | 0.444 | 0.029 |
| Multiple Imputation^2^ |  |  | -0.3 | -0.2 | -1.0 to 0.5 | 0.593 | 0.030 |
| Complete Case |  |  | -0.7 | -0.6 | -1.5 to 0.3 | 0.183 | 0.036 |
| **School ecology/climate (SCCS**) |  |  |  |  |  |  |  |
| School Leadership and Student Involvement |  |  |  |  |  |  |  |
| Multiple Imputation^1^ | N = 1472  3.1 (0.9) | N = 1417  3.1 (0.8) | 0.02 | 0.003 | -0.1 to 0.1 | 0.965 | 0.092 |
| Multiple Imputation^2^ |  |  | 0.04 | 0.02 | -0.1 to 0.2 | 0.807 | 0.106 |
| Complete Case |  |  | 0.003 | 0.03 | -0.1 to 0.2 | 0.729 | 0.098 |
| Respectful Climate |  |  |  |  |  |  |  |
| Multiple Imputation^1^ | N = 1469  3.3 (0.7) | N = 1417  3.3 (0.7) | 0.02 | 0.01 | -0.1 to 0.1 | 0.837 | 0.059 |
| Multiple Imputation^2^ |  |  | 0.03 | 0.02 | -0.1 to 0.1 | 0.770 | 0.076 |
| Complete Case |  |  | 0.01 | 0.03 | -0.1 to 0.2 | 0.687 | - |
| Peer Climate |  |  |  |  |  |  |  |
| Multiple Imputation^1^ | N = 1468  3.0 (0.7) | N = 1417  3.1 (0.8) | 0.01 | 0.001 | -0.1 to 0.1 | 0.982 | 0.076 |
| Multiple Imputation^2^ |  |  | 0.01 | -0.004 | -0.1 to 0.1 | 0.954 | 0.090 |
| Complete Case |  |  | -0.01 | -0.005 | -0.1 to 0.1 | 0.941 | 0.093 |
| Caring Adults |  |  |  |  |  |  |  |
| Multiple Imputation^1^ | N = 1467  3.1 (0.9) | N = 1416  3.1 (0.9) | 0.03 | 0.04 | -0.1 to 0.1 | 0.512 | 0.048 |
| Multiple Imputation^2^ |  |  | 0.1 | 0.1 | -0.1 to 0.2 | 0.306 | 0.060 |
| Complete Case |  |  | 0.02 | 0.05 | -0.1 to 0.2 | 0.390 | 0.048 |

^1^ The multiple imputation model included outcomes at baseline (where available), as well as all four post-baseline timepoints. Utility scores were included in the imputation model, and gender was maintained as three categories - “female”, “male” & “other” for imputation and analysis.

^2^ The multiple imputation model included only outcomes at baseline (where available), pre-intervention and at two-years. Utility scores were excluded from the imputation model, and gender was dichotomized into “female” versus “male & other” for imputation and analysis.

^a^ Odds ratios are presented for the outcomes: drug use (any drug use versus no drug use), alcohol use (any alcohol use versus no alcohol use), self harm (“yes” & ”prefer not to say” versus “no”) and suicide ideation (“yes” & “prefer not to say” versus “no”).

^b^ ICCs from unadjusted analyses.

Note: The Ns presented in the table represent the number of students providing valid outcome data for each measurement.

SBMT: school-based mindfulness training. TAU: teaching as usual. ICC: intra-cluster correlation coefficient. SD: standard deviation. CI: confidence interval. BRIEF2: Behaviour Rating Inventory of Executive Function, Second edition. RCADS: Revised Child Anxiety and Depression Scale. CAMM: Child-and Adolescent Mindfulness Measure. SCCS: School Climate and Connectedness survey.

**Supplementary Table S4:** Results from main and additional analyses of teacher outcomes at two-years: Cohort 1

| **Outcome** | **SBMT** | **TAU** | **Unadjusted** | **Adjusted mean difference** | | | **ICC^a^** |
| --- | --- | --- | --- | --- | --- | --- | --- |
|  | **mean (SD)** | **mean (SD)** | **mean difference** | **estimate** | **95% CI** | **p-value** |  |
| **Well-being – Maslach Burn-out Inventory (MBI)** | | | | | | | |
| Exhaustion |  |  |  |  |  |  |  |
| Multiple Imputation | N = 31  2.0 (1.1) | N = 22  2.7 (1.4) | -0.8 | -0.8 | -1.9 to 0.4 | 0.193 | 0.14 |
| Complete Case |  |  | -0.9 | -0.8 | -1.8 to 0.2 | 0.123 | 0.21 |
| CACE |  |  | -2.3 | -2.2 | -5.2 to 0.8 | 0.143 | - |
| Depersonalisation |  |  |  |  |  |  |  |
| Multiple Imputation | N = 31  0.7 (0.7) | N = 22  1.4 (1.5) | -0.5 | 0.1 | -0.7 to 1.0 | 0.782 | 0.12 |
| Complete Case |  |  | -0.7 | -0.3 | -0.7 to 0.2 | 0.245 | 0.03 |
| CACE |  |  | -1.4 | 0.2 | -1.7 to 2.2 | 0.819 | - |
| Personal Accomplishment |  |  |  |  |  |  |  |
| Multiple Imputation | N = 31  1.0 (0.7) | N = 22  1.1 (0.8) | 0.2 | 0.3 | -0.4 to 1.1 | 0.346 | 0.15 |
| Complete Case |  |  | -0.1 | -0.02 | -0.4 to 0.4 | 0.945 | 0.15 |
| CACE |  |  | 0.6 | 0.2 | -1.2 to 1.5 | 0.819 | - |
| **Self-efficacy questionnaire** | | | | | | | |
| Student Engagement subscale |  |  |  |  |  |  |  |
| Multiple Imputation | N = 29  6.9 (1.1) | N = 22  6.3 (1.5) | 0.4 | -0.2 | -1.3 to 0.9 | 0.747 | 0.11 |
| Complete Case |  |  | 0.7 | 0.04 | -0.7 to 0.8 | 0.910 | 0.30 |
| CACE |  |  | 0.8 | 0.7 | -1.6 to 3.0 | 0.548 | - |
| Instructional Practice Subscale |  |  |  |  |  |  |  |
| Multiple Imputation | N = 29  7.4 (1.2) | N = 22  7.4 (1.2) | 0.3 | 0.3 | -0.9 to 1.4 | 0.672 | 0.26 |
| Complete Case |  |  | 0.1 | 0.01 | -1.0 to 1.0 | 0.983 | 0.32 |
| CACE |  |  | 0.6 | 1.1 | -1.0 to 3.3 | 0.304 | - |
| Classroom Management Subscale |  |  |  |  |  |  |  |
| Multiple Imputation | N = 29  7.5 (1.2) | N = 22  7.3 (1.2) | 0.7 | 0.5 | -0.4 to 1.3 | 0.279 | 0.17 |
| Complete Case |  |  | 0.3 | 0.2 | -0.2 to 0.7 | 0.367 | 0.23 |
| CACE |  |  | 1.6 | 1.4 | -0.4 to 3.2 | 0.141 | - |
| **Mindfulness (FFMQ-SF)** |  |  |  |  |  |  |  |
| Multiple Imputation | N = 30  90.3 (13.5) | N = 22  84.6 (16.8) | 2.8 | -0.6 | -10.5 to 9.4 | 0.913 | 0.17 |
| Complete Case |  |  | 7.2 | 3.8 | -7.7 to 15.3 | 0.520 | 0.28 |
| CACE |  |  | 5.4 | 1.6 | -20.7 to 23.9 | 0.890 | - |
| **Mindfulness (MTS) - Interpersonal** |  |  |  |  |  |  |  |
| Multiple Imputation | N = 31  20.8 (2.0) | N = 22  20.3 (2.5) | 1.1 | 1.1 | -1.3 to 3.5 | 0.352 | 0.17 |
| Complete Case |  |  | 0.5 | 0.7 | -0.9 to 2.4 | 0.388 | 0.09 |
| CACE |  |  | 2.5 | 3.5 | -0.8 to 7.8 | 0.113 | - |
| **Mindfulness (MTS) - Intrapersonal** |  |  |  |  |  |  |  |
| Multiple Imputation | N = 30  32.6 (5.9) | N = 22  32.7 (6.9) | -1.0 | -2.1 | -7.6 to 3.4 | 0.452 | 0.23 |
| Complete Case |  |  | 0.7 | 0.2 | -4.3 to 4.7 | 0.938 | 0.30 |
| CACE |  |  | -5.6 | 0.5 | -10.0 to 10.9 | 0.932 | - |
| **Stress (PSS)** |  |  |  |  |  |  |  |
| Multiple Imputation | N = 31  14.0 (7.9) | N = 22  15.8 (9.5) | -0.2 | 1.3 | -4.8 to 7.3 | 0.676 | 0.11 |
| Complete Case |  |  | -2.5 | -0.9 | -7.2 to 5.3 | 0.770 | 0.20 |
| CACE |  |  | 1.2 | 2.8 | -12.1 to 17.7 | 0.712 | - |
| **Depression (PHQ-9)** |  |  |  |  |  |  |  |
| Multiple Imputation | N = 31  4.3 (4.2) | N = 22  5.2 (4.9) | 0.8 | 1.2 | -1.6 to 4.1 | 0.394 | 0.04 |
| Complete Case |  |  | -1.1 | 0.2 | -3.1 to 3.5 | 0.917 | 0.19 |
| CACE |  |  | 2.6 | 2.7 | -3.0 to 8.5 | 0.347 | - |
| **Anxiety (GAD-7)** |  |  |  |  |  |  |  |
| Multiple Imputation | N = 31  3.5 (3.4) | N = 22  5.0 (5.2) | -0.6 | 0.1 | -2.4 to 2.5 | 0.964 | 0.06 |
| Complete Case |  |  | -1.6 | -0.7 | -3.6 to 2.1 | 0.620 | 0.08 |
| CACE |  |  | -1.4 | 0.5 | -5.1 to 6.2 | 0.852 | - |
| **School Ecology/Climate (SCCS)** |  |  |  |  |  |  |  |
| School leadership and involvement | | | | | | | |
| Multiple Imputation | N = 28  3.9 (0.7) | N = 22  3.9 (0.7) | 0.4 | 0.4 | -0.4 to 1.1 | 0.310 | 0.19 |
| Complete Case |  |  | -0.02 | -0.1 | -0.4 to 0.2 | 0.515 | 0.07 |
| CACE |  |  | 1.2 | 1.1 | -0.3 to 2.5 | 0.115 | - |
| Staff Attitudes |  |  |  |  |  |  |  |
| Multiple Imputation | N = 28  4.1 (0.6) | N = 22  4.3 (0.4) | 0.4 | -0.6 | -1.3 to 0.1 | 0.091 | 0.18 |
| Complete Case |  |  | -0.3 | -0.3 | -0.7 to 0.03 | 0.071 | 0.36 |
| CACE |  |  | -1.8 | -0.9 | -2.5 to 0.6 | 0.244 | - |
| Respectful Climate |  |  |  |  |  |  |  |
| Multiple Imputation | N = 28  3.6 (0.6) | N = 22  3.7 (0.6) | 0.1 | 0.1 | -0.7 to 0.9 | 0.871 | 0.31 |
| Complete Case |  |  | -0.1 | -0.03 | -0.4 to 0.3 | 0.896 | 0.14 |
| CACE |  |  | 0.2 | 0.4 | -1.1 to 1.9 | 0.603 | - |

^a^ ICCs from unadjusted analyses.

SBMT: school-based mindfulness training. TAU: teaching as usual. ICC: intra-cluster correlation coefficient. SD: standard deviation. CI: confidence interval. CACE: complier average causal effect. FFMQ-SF: Five Facet Mindfulness Questionnaire Short Form. MTS: Mindfulness in Teaching Scale. PSS: Perceived Stress Scale. PHQ-9: Patient Health Questionnaire. GAD-7: Generalised Anxiety Disorder assessment.

**Supplementary Table S5:** Results from main and additional analyses of teacher outcomes at two-years: Cohort 2

| **Outcome** | **SBMT** | **TAU** | **Unadjusted** | **Adjusted mean difference** | | | **ICC^a^** |
| --- | --- | --- | --- | --- | --- | --- | --- |
|  | **mean (SD)** | **mean (SD)** | **mean difference** | **estimate** | **95% CI** | **p-value** |  |
| **Well-being – Maslach Burn-out Inventory (MBI)** | | | | | | | |
| Exhaustion |  |  |  |  |  |  |  |
| Multiple Imputation | N = 153  2.3 (1.2) | N = 123  2.3 (1.1) | -0.1 | -0.1 | -0.4 to 0.1 | 0.324 | 0.06 |
| Complete Case |  |  | 0.1 | -0.01 | -0.2 to 0.2 | 0.899 | 0.004 |
| CACE |  |  | -0.2 | -0.4 | -1.0 to 0.2 | 0.202 | - |
| Depersonalisation |  |  |  |  |  |  |  |
| Multiple Imputation | N = 153  0.8 (0.7) | N = 123  0.8 (0.9) | -0.1 | -0.1 | -0.3 to 0.1 | 0.183 | 0.09 |
| Complete Case |  |  | -0.02 | -0.1 | -0.2 to 0.1 | 0.554 | 0.00 |
| CACE |  |  | -0.4 | -0.5 | -1.0 to 0.1 | 0.090 | - |
| Personal Accomplishment (lack of) |  |  |  |  |  |  |  |
| Multiple Imputation | N = 153  1.0 (0.7) | N = 123  1.1 (0.9) | -0.2 | -0.2 | -0.3 to -0.001 | 0.049 | 0.04 |
| Complete Case |  |  | -0.1 | -0.2 | -0.3 to -0.02 | 0.025 | 0.00 |
| CACE |  |  | -0.5 | -0.5 | -0.9 to -0.1 | 0.015 | - |
| **Self-efficacy questionnaire** | | | | | | | |
| Student Engagement subscale |  |  |  |  |  |  |  |
| Multiple Imputation | N = 149  7.1 (1.0) | N = 117  6.9 (1.0) | 0.2 | 0.2 | -0.04 to 0.4 | 0.123 | 0.09 |
| Complete Case |  |  | 0.2 | 0.1 | -0.1 to 0.4 | 0.180 | 0.05 |
| CACE |  |  | 0.5 | 0.5 | -0.05 to 1.1 | 0.072 | - |
| Instructional Practice Subscale |  |  |  |  |  |  |  |
| Multiple Imputation | N = 149  7.6 (0.9) | N = 117  7.5 (0.9) | -0.005 | 0.03 | -0.2 to 0.2 | 0.752 | 0.10 |
| Complete Case |  |  | 0.1 | 0.1 | -0.1 to 0.3 | 0.281 | 0.04 |
| CACE |  |  | 0.01 | 0.1 | -0.4 to 0.7 | 0.657 | - |
| Classroom Management Subscale |  |  |  |  |  |  |  |
| Multiple Imputation | N = 149  7.8 (0.9) | N = 117  7.6 (0.9) | 0.2 | 0.2 | -0.02 to 0.4 | 0.079 | 0.01 |
| Complete Case |  |  | 0.2 | 0.1 | -0.1 to 0.3 | 0.313 | 0.00 |
| CACE |  |  | 0.6 | 0.5 | -0.02 to 1.1 | 0.057 | - |
| **Mindfulness (FFMQ-SF)** |  |  |  |  |  |  |  |
| Multiple Imputation | N = 150  85.4 (12.8) | N = 117  86.4 (12.9) | -0.6 | 0.5 | -1.9 to 2.9 | 0.675 | 0.06 |
| Complete Case |  |  | -1.0 | 0.1 | -2.5 to 2.8 | 0.936 | 0.00 |
| CACE |  |  | -1.6 | 2.6 | -3.4 to 8.6 | 0.384 | - |
| **Mindfulness (MTS) - Interpersonal** |  |  |  |  |  |  |  |
| Multiple Imputation | N = 151  21.3 (2.3) | N = 119  21.0 (2.5) | 0.2 | 0.2 | -0.3 to 0.7 | 0.401 | 0.01 |
| Complete Case |  |  | 0.3 | 0.3 | -0.2 to 0.8 | 0.251 | 0.00 |
| CACE |  |  | 0.6 | 0.7 | -0.5 to 1.9 | 0.267 | - |
| **Mindfulness (MTS) - Intrapersonal** |  |  |  |  |  |  |  |
| Multiple Imputation | N = 151  32.6 (4.8) | N = 119  33.9 (4.9) | -1.1 | -0.6 | -1.6 to 0.3 | 0.187 | 0.02 |
| Complete Case |  |  | -1.3 | -0.6 | -1.5 to 0.4 | 0.258 | 0.03 |
| CACE |  |  | -3.2 | -1.5 | -4.0 to 1.0 | 0.230 | - |
| **Stress (PSS)** |  |  |  |  |  |  |  |
| Multiple Imputation | N = 151  15.7 (7.3) | N = 118  16.4 (6.1) | -1.6 | -1.6 | -3.0 to -0.3 | 0.020 | 0.03 |
| Complete Case |  |  | -0.7 | -1.0 | -2.4 to 0.5 | 0.198 | 0.00 |
| CACE |  |  | -4.6 | -4.6 | -8.2 to -1.1 | 0.011 | - |
| **Depression (PHQ-9)** |  |  |  |  |  |  |  |
| Multiple Imputation | N = 151  5.3 (4.4) | N = 118  5.2 (3.7) | -0.3 | -0.3 | -1.2 to 0.7 | 0.562 | 0.01 |
| Complete Case |  |  | 0.1 | -0.2 | -1.1 to 0.8 | 0.733 | 0.00 |
| CACE |  |  | -0.8 | -1.2 | -3.6 to 1.3 | 0.342 | - |
| **Anxiety (GAD-7)** |  |  |  |  |  |  |  |
| Multiple Imputation | N = 150  4.8 (4.3) | N =117  4.3 (3.5) | -0.4 | -0.1 | -1.0 to 0.7 | 0.746 | 0.02 |
| Complete Case |  |  | 0.5 | 0.2 | -0.6 to 1.0 | 0.635 | 0.00 |
| CACE |  |  | -1.1 | -0.8 | -2.9 to 1.4 | 0.489 | - |
| **School Ecology/Climate (SCCS)** |  |  |  |  |  |  |  |
| School Leadership and Involvement | | | | | | | |
| Multiple Imputation | N =151  4.0 (0.7) | N = 124  3.9 (0.6) | 0.2 | 0.2 | 0.02 to 0.3 | 0.028 | 0.16 |
| Complete Case |  |  | 0.1 | 0.1 | -0.02 to 0.3 | 0.096 | 0.16 |
| CACE |  |  | 0.5 | 0.5 | 0.1 to 0.9 | 0.017 | - |
| Staff Attitudes |  |  |  |  |  |  |  |
| Multiple Imputation | N = 151  4.2 (0.6) | N = 124  4.1 (0.5) | 0.1 | 0.2 | 0.03 to 0.4 | 0.023 | 0.20 |
| Complete Case |  |  | 0.1 | 0.1 | -0.03 to 0.2 | 0.130 | 0.13 |
| CACE |  |  | 0.3 | 0.5 | 0.1 to 1.0 | 0.012 | - |
| Respectful Climate |  |  |  |  |  |  |  |
| Multiple Imputation | N = 151  3.9 (0.5) | N = 124  3.7 (0.6) | 0.2 | 0.2 | 0.1 to 0.4 | 0.001 | 0.23 |
| Complete Case |  |  | 0.2 | 0.2 | 0.04 to 0.3 | 0.011 | 0.23 |
| CACE |  |  | 0.6 | 0.6 | 0.2 to 1.0 | 0.002 | - |

^a^ ICCs from unadjusted analyses.

SBMT: school-based mindfulness training. TAU: teaching as usual. ICC: intra-cluster correlation coefficient. SD: standard deviation. CI: confidence interval. CACE: complier average causal effect. FFMQ-SF: Five Facet Mindfulness Questionnaire Short Form. MTS: Mindfulness in Teaching Scale. PSS: Perceived Stress Scale. PHQ-9: Patient Health Questionnaire. GAD-7: Generalised Anxiety Disorder assessment.
